# Supplementary figures and images for: Growth hormone increases regulator of calcineurin 1-4 (Rcan1-4) mRNA through c-JUN in rat liver
Source: PLoS One. 2020 Jun 26;15(6):e0235270. doi: 10.1371/journal.pone.0235270 (PMC7319343; doi:10.1371/journal.pone.0235270)

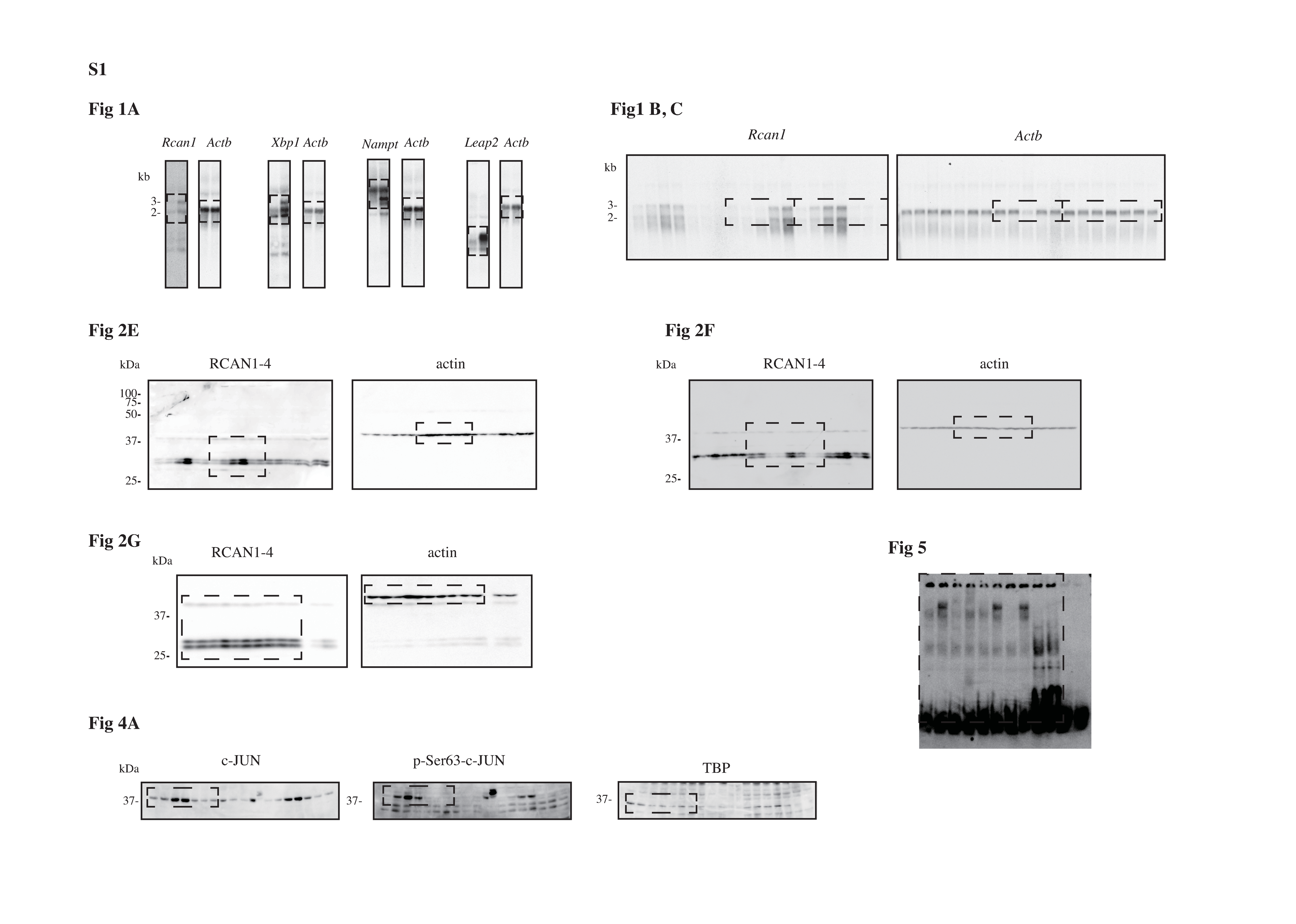

Supplement: S1 Raw images — (TIF) [file pone.0235270.s003.tif]
